# Synchronous Environmental and Cultural Change in the Emergence of Agricultural Economies 10,000 Years Ago in the Levant

**DOI:** 10.1371/journal.pone.0134810

**Published:** 2015-08-04

**Authors:** Ferran Borrell, Aripekka Junno, Joan Antón Barceló

**Affiliations:** 1 Centre de recherche français à Jérusalem (CRFJ-CNRS), Jerusalem, Israel; 2 Department of Philosophy, History, Culture and Art Studies, University of Helsinki, Helsinki, Finland; 3 Department of Prehistory, Universitat Autònoma de Barcelona, Barcelona, Spain; Université de Poitiers, FRANCE

## Abstract

The commonly held belief that the emergence and establishment of farming communities in the Levant was a smooth socio-economic continuum during the Pre-Pottery Neolithic (ca. 12,000-9,000 cal BP) with only rare minor disruptions is challenged by recently obtained evidence from this region. Using a database of archaeological radiocarbon dates and diagnostic material culture records from a series of key sites in the northern Levant we show that the hitherto apparent long-term continuity interpreted as the origins and consolidation of agricultural systems was not linear and uninterrupted. A major cultural discontinuity is observed in the archaeological record around 10,000 cal BP in synchrony with a Holocene Rapid Climate Change (RCC), a short period of climatic instability recorded in the Northern Hemisphere. This study demonstrates the interconnectedness of the first agricultural economies and the ecosystems they inhabited, and emphasizes the complex nature of human responses to environmental change during the Neolithic period in the Levant. Moreover, it provides a new environmental-cultural scenario that needs to be incorporated in the models reconstructing both the establishment of agricultural economy in southwestern Asia and the impact of environmental changes on human populations.

## Introduction

The ‘Fertile Crescent’ is considered both the homeland of Near Eastern agriculture and the cradle of the ancient Near Eastern civilizations. The origins and consolidation of agricultural practices in the Levant and their subsequent diffusion throughout the western Mediterranean basin (12^th^-7^th^ millennia cal BP) had a profound impact on human society, irrevocably altering social interaction between communities and the relationship of people with their natural surroundings [1–7]. This process, often referred to by the term ‘Neolithic Revolution’, took place at the beginning of the Holocene period, during the Pre-Pottery Neolithic (Table 1). It is characterized by the rapid emergence of genuine village societies throughout a broad cultural interaction zone extending over the entire Levant and beyond [1,2].

**Table 1:** Approximate chronology of the Pre-Pottery Neolithic in the Levant.

Northern Levant	cal BP	Southern Levant	cal BP
PPNA	12,200–10,800	PPNA	11,600–10,500
Early PPNB	10,800–10,200	Early PPNB	10,500–10,100
Middle PPNB	10,200–9,600	Middle PPNB	10,100–9,500
Late PPNB	9,600–8,900	Late PPNB	9,500–8,750
Pottery Neolithic/Final PPNB	8,900–8,400	Final PPNB/PPNC	8,750–8,400
		Early Pottery Neolithic	8,400–7,600

During the early stages of the Neolithic, known as Pre-Pottery Neolithic A (abbreviated as PPNA; *ca*. 12,200/11,600–10,800/10,500 cal BP), archaic village society developed with the introduction of cultivation, supplemented by continued foraging and hunting. A broad range of animals and especially plant resources was systematically exploited as population size increased, with the subsequent impact upon social relations and exchange networks. Indeed, early cultivation has been identified at a series of PPNA sites from both the northern and southern Levant [8,9]. The steady rise of temperature and humidity after the Younger Dryas event ~11,600 cal BP [10] involved an increase in rainfall and probably affected the availability of food plants, which became a contributing factor to the emergence of agriculture in the Near East [11,12]. The Pre-Pottery Neolithic B (abbreviated as PPNB and further subdivided into Early-Middle-Late; *ca*. 10,800/10,500–9,000/8,400 cal BP) represents the emergence of complex village societies throughout the Levant (including the ‘megasite’ phenomenon in Jordan [13–15]), associated with farming, increased sedentism and animal domestication, during a period of relative climate stability. Full-fledged agriculture of domesticated cereals and legumes is dated in the Levant to about 10,000 cal BP [8,9,16]. This change entailed a quantum leap in population size and social interaction and was qualitatively and quantitatively more complex than during the preceding periods [1,2,17–20].

**Fig 1:**
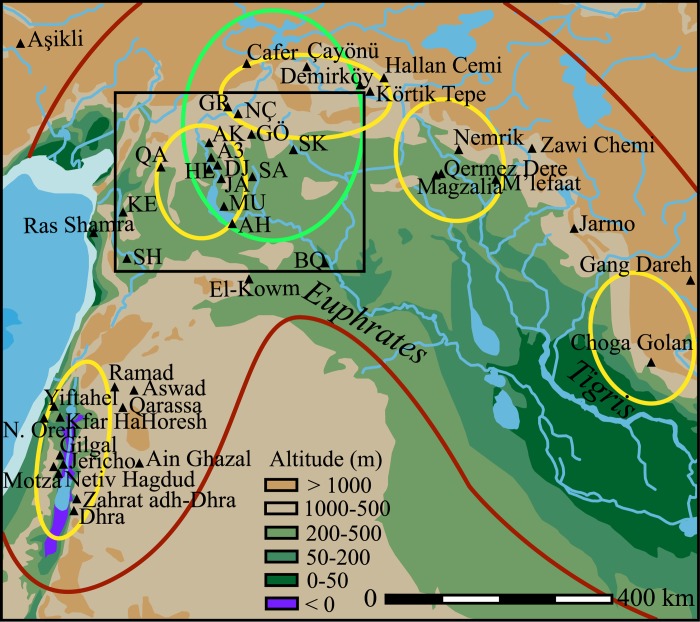
Location of the study region (black square) and some key Neolithic sites. The red line delimits the Fertile Crescent; the green circle delimits the single core region according to some authors [3]; and the yellow circles indicate the different core regions according to others [7]. Note that due to the density of sites in the study region, names have been abbreviated: SH = Shir; KE = Ain el-Kerkh; QA = Qaramel; GR = Gritille; AK = Akarçay; A3 = ‘Abr 3; HL = Halula; DJ = Dja’de; JA = Jerf el Ahmar; MU = Mureybet; AH = Abu Hureyra; BO = Bouqras; NÇ = Nevalı Çori; GÖ = Göbekli; SK = Seker al-Aheimar; and SA = Sabi Abyad.

The origins and nature of such processes that developed during two millennia (12,000–10,000 cal BP) became a subject for intense discussions, mostly focused on the identification of the earliest cultivation of the wild progenitors of the founder crops. Some researchers argue for a single regional location for the initiation of cultivation in south-east Turkey and the middle Euphrates valley followed by subsequent dispersions throughout the Near East [2,3,21,22], while others [7,9,14,23–25] assume polycentric developments (Fig 1). It is noteworthy that both paradigms accept the key role of the early Holocene rise in temperature and humidity in the distribution and development of new ecosystems that favoured the appearance of agriculture in the Levant.

In the northern Levant, the middle Euphrates valley and its neighbouring regions in particular, the excavations at a series of single-phased and multiple-phased Neolithic settlements manifest apparent cultural continuity throughout the PPNA and PPNB periods. This broadly assumed uninterrupted sequence during the entire Pre-Pottery Neolithic has greatly contributed to conceiving the Neolithization process in the Levant as an incremental continuum with relatively minor disruptions. However, a recent study of radiocarbon dates and a detailed analysis of PPN bidirectional blade production strategies throughout the northern Levant indicates an interruption in the sequence of settlement in the middle Euphrates valley *ca*. 10,200–9,800 cal BP followed by a substantial cultural transformation, as suggested by important changes in blade production strategies from the PPNA and Early PPNB through to the Middle and Late PPNB [26].

The present paper aims to verify the above statement and analyze both the causes and consequences for the cultural break. In order to avoid partial interpretations based on a single discipline we integrate detailed multi-proxy archaeological records (e.g. radiocarbon dating, settlement patterns, symbolism, flint knapping traditions and archaeobotany) with available palaeoclimate proxies from both the Eastern Mediterranean and the North Atlantic. Data on animal domestication has not been included. Active management of all four major livestock species is attested in the Levant *ca*. 11,000 to 10,000 cal BP and clear-cut morphological responses to domestication are evident *ca*. 9,500 to 9,000 cal BP [27,28]. However, unlike plants, selective pressures on animals undergoing domestication may only be indirectly linked to archaeologically detectable morphological changes [29], making identification of animal domestication even more difficult.

Finally, the analysis is intentionally focused on the unique and distinctive archaeological record of the middle Euphrates valley and its neighbouring regions. The reason for this geographic focus is first because this region is the centre of the debate concerning the emergence of farming in southwestern Asia. Second, it has provided a coherent and culturally homogeneous regional picture that has benefited from long-term international research projects comprising intensive field work at a series of key sites. Third, several decades of research indicate that there is no evidence for large-scale geomorphologic and post-depositional processes affecting the quality and reliability of the archaeological record.

## Results

### Sites and ^14^C chronology

The most relevant sites for understanding the Levantine Neolithic, such as Mureybet, Göbekli or Halula, are located in the basin of the Euphrates River, a region with a high density of sites that in most cases are multi-phased and cover time spans of several hundreds of years. These sites represent all the recognized phases of the Pre-Pottery Neolithic indicating an enduring continuity of established agriculture and herding subsistence from its beginnings through its cultural consolidation and intensification in the middle Euphrates valley, and by extension, across the northern Levant.

**Fig 2:**
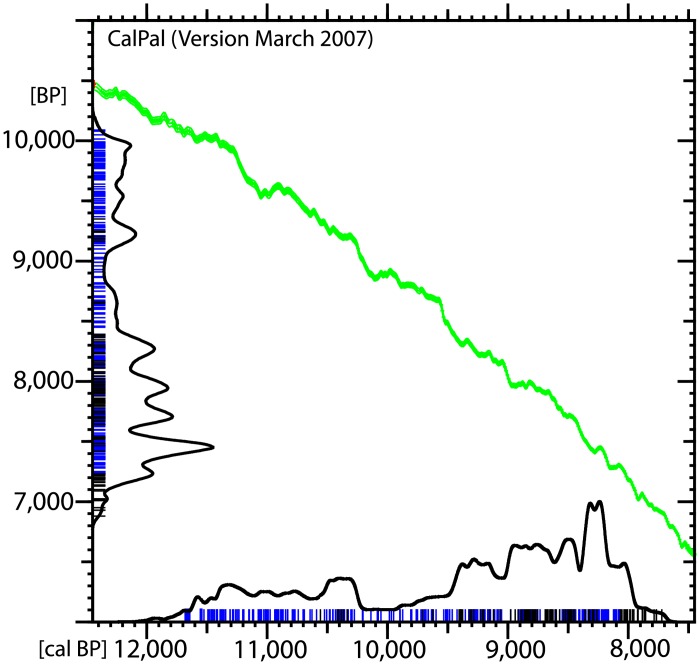
Summed probability distribution of 450 ^14^C dates. Generated in CalPal [30] using IntCal04 calibration curve.

The analysis of a series of 450 calibrated radiocarbon dates (see S1 Table for description of each sample) covering a span of ~3000 years, between 11,700 and 8,700 cal BP, demonstrates a significant reduction in the volume of archaeological signal around 10,000 cal BP (Fig 2). Such a decrease indicates that the apparently uninterrupted sequence of settlements from PPNA to PN cannot be taken as unequivocal evidence of settlement continuity. It appears that the period comprised *ca*. 10,200–9,800 cal BP is poorly represented, indicating a break in the settlement pattern probably marked by a decrease in population density.

**Fig 3:**
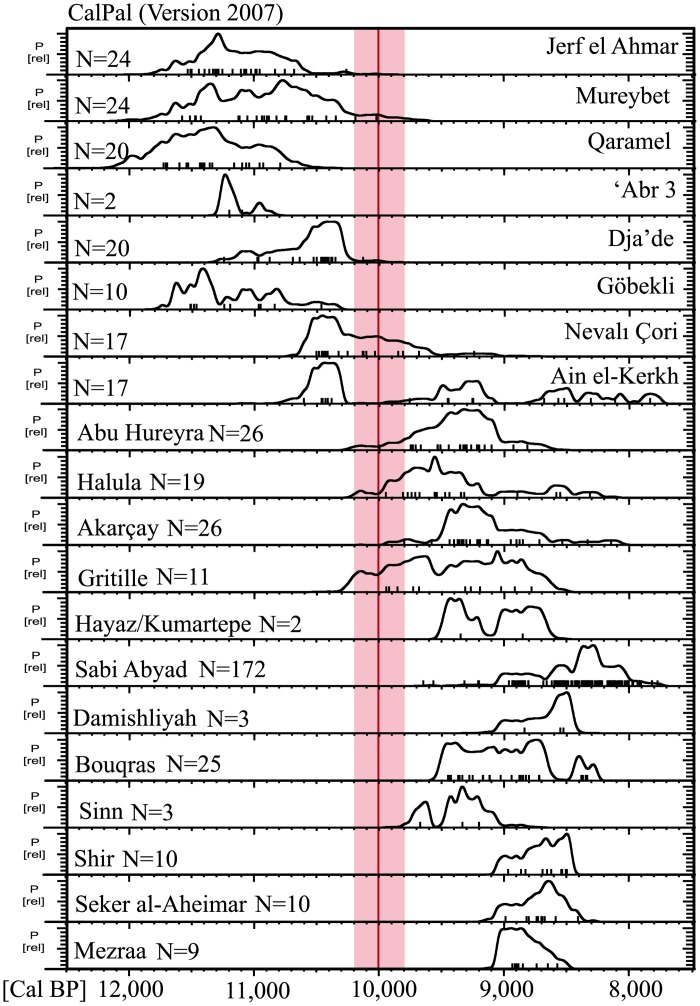
Summed probability distribution of each settlement analyzed. Generated in CalPal [30] using IntCal04 calibration curve.

**Fig 4:**
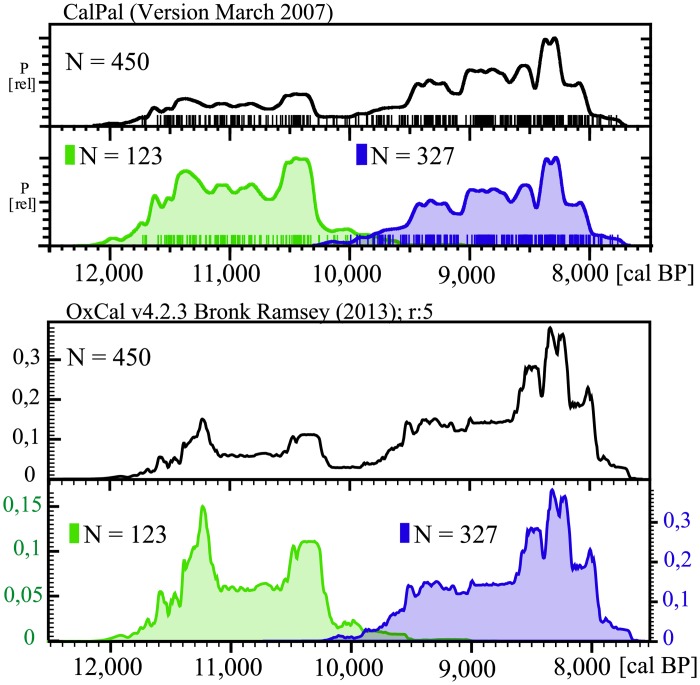
Summed probability distribution of 450 ^14^C dates. Generated in CalPal [30] using the IntCal04 calibration curve and in OxCal [31] using the IntCal13 calibration curve (upper segments) and summed probability distribution of both settlement groups (coloured in green and blue) analyzed separately but displayed together in the same chart for comparison (lower segments). Note that even though computer implementations are different, the results are the same, strengthening the reliability of our observations.

Comparison of the summed probability distribution of ^14^C dates for each site indicates the presence of a break in the regional settlement pattern and a clear disruption of occupation between the old and the newly established sites among the two clusters (Fig 3). None of the settlements in the region dating to the second half of the 12^th^ through the 11^th^ millennia was occupied at *ca*. 9,800 cal BP. Mureybet, Jerf el Ahmar, Dja’de, ‘Abr, Göbekli and Qaramel were all abandoned before *ca*. 10,000 cal BP. In the upper part of the middle Euphrates valley, only Nevalı Çori shows evidence of having been occupied by *ca*. 10,200–9,800 cal BP. Interestingly, most evidence from this site is dated to the third quarter of the 11^th^ millennium cal BP and little is known about its later occupation, just before it was abandoned by *ca*. 9,800 cal BP. This indicates that Nevalı Çori and Gürçütepe (the latter not dated but attributed to the Late PPNB) were not contemporary. The site of Ain el Kerkh is paradigmatic, displaying a disruption of almost 500 years between the occupation dated to the Early PPNB and the later Late PPNB habitation that fully accords with the phenomenon observed at the regional scale. The second cluster of Neolithic sites in the region comprise Akarçay, Mezraa Teleilat, Gritille, Hayaz, Kumartepe, Gürçütepe, Sabi Abyad, Damishliyah, Seker al-Aheimar, Halula, Abu Hureyra, Bouqras, Sinn and Shir, all newly founded not earlier than 9,800 cal BP or even later. The break in the settlement pattern and the phenomenon of settlement/population replacement is also apparent when comparing the summed probability distribution of both groups of settlements (Fig 4). The two series of age determinations barely overlap, with a clear settlement break at around 10,200–9,800 cal BP, indicating that none of the earlier settlements played a significant role in the later appearance of a new series of settlements. In other words, the rupture defines two non-contiguous archaeological periods during the Pre-Pottery Neolithic separated by a prolonged hiatus.

In conclusion, the reduced archaeological signal might indicate that during a short period of time this region had a very low population density, or was even abandoned, and thus the process of successful agro-pastoral development in the northern Levant was not linear and uninterrupted [26]. Moreover, the hiatus in settlement (10,200–9,800 cal BP) covers almost the totality of the time span traditionally attributed to the Middle PPNB (10,200–9,600 cal BP), which becomes almost devoid of archaeological material and quite inconsistent as a chrono-cultural period.

### Settlement discontinuity and changing settlement patterns: the end of a myth

**Fig 5:**
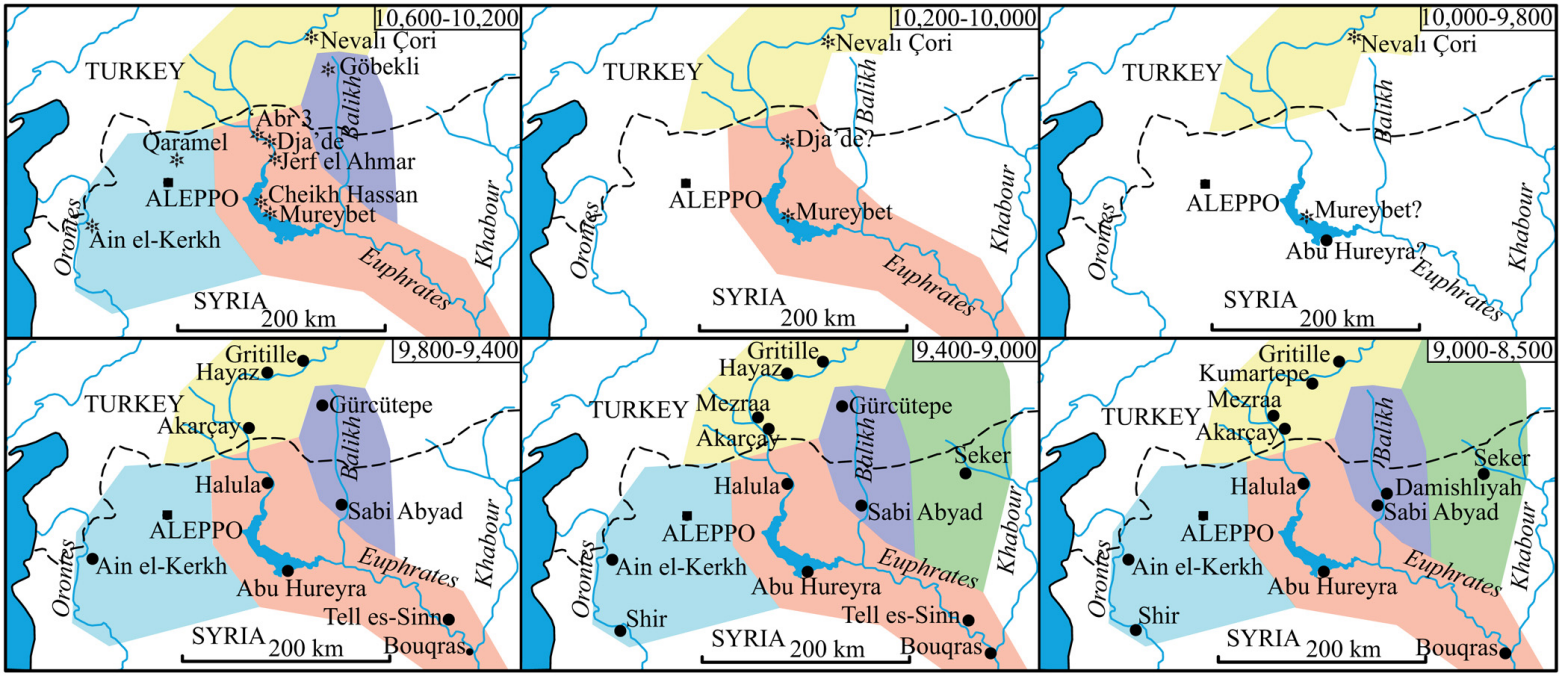
Settlement patterns. The history of the settlements in the studied region from *ca*. 10,600 to 8,500 cal BP.

The settlement break indicated by the summed probability distribution of calibrated ^14^C dates is observed in all sub-areas of the study region (e.g. the entire middle Euphrates valley, upper Khabur valley, Balikh valley, the Jazireh, and Orontes valley) and apparently none of these sub-regions served as stable refugia for the inhabitants of the earlier settlements (Fig 5). Furthermore, with the exception of Ain el Kerkh, the new settlements that were established from 9,800 cal BP onwards were founded in different locations. Thus, the locations of the earlier settlements were avoided by the new settlers whose strategic choices for site locations differed from those of the earlier populations in this region.

**Fig 6:**
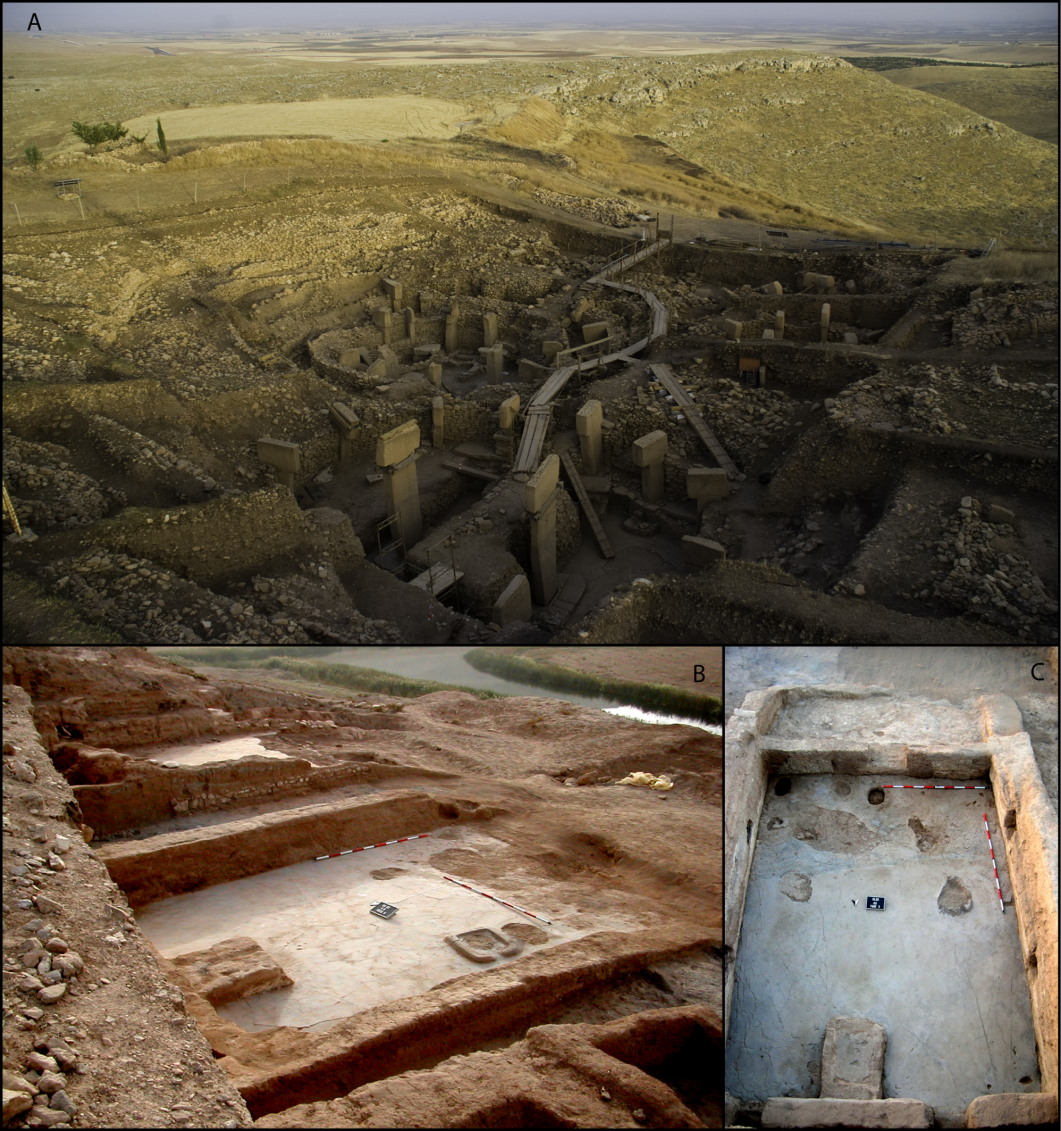
Settlement type, architecture and internal organization. Note the major differences between Göbekli (above) and Halula (below).

In addition, it is not only the location but the site-size and site-organization that differ. With the exception of Göbekli, the earlier Neolithic settlements (*ca*. 11,500–10,200 cal BP) are relatively small (e.g. Mureybet—3.5 ha, Dja’de—1.5 ha; Nevalı Çori >1 ha). Concerning site-organization, these settlements were also characterized by the monumental aspect of their architecture (some of which served as cult centres of supra-regional importance), and by well-developed, rich and specific symbolic features (Fig 6A). Collective buildings and their associated specific symbolic features seem to have played a key role in the economic organization of the community. However, none of these features show obvious continuity in the newly founded settlements after the break, a phenomenon that has already been pointed out by some authors [32,33] and interpreted as a real change in symbolic activities. The new villages were founded in different locations and are larger than the earlier ones (e.g. Akarcay—5 ha, Halula—8 ha, Bouqras—5 ha, Abu Hureyra—12 ha and Gürçütepe—10 ha). In most cases the internal organization of houses and installations is fairly packed, comprising numerous middle-sized rectangular domestic buildings, often displaying very standardized architectural patterns (e.g. Halula), which could be interpreted as family households (Fig 6B and 6C). These later settlements represent a clear shift towards larger sedentary villages that lasted for many centuries and demonstrate continuity from the Pre-Pottery to the Pottery Neolithic, with the household as the fundamental unit of its socio-economic organization [20].

### Bidirectional Blade Technology: doing old things in a new way

During the Pre-Pottery Neolithic, bidirectional blade technology formed the basis of flaked-stone industries throughout the Levant. Lithic studies focused on bidirectional technology have been able to identify cultural attributes of the first farming communities in the Near East, such as social complexity, inter-site and intra-site social interactions, knowledge transfer, exchange networks and product circulation. Furthermore, they have permitted the reconstruction of the reduction sequences and the technological skills and behavioural patterns of their users, evidencing temporal and spatial variability in employing the bidirectional technology during that period [34–37].

**Fig 7:**
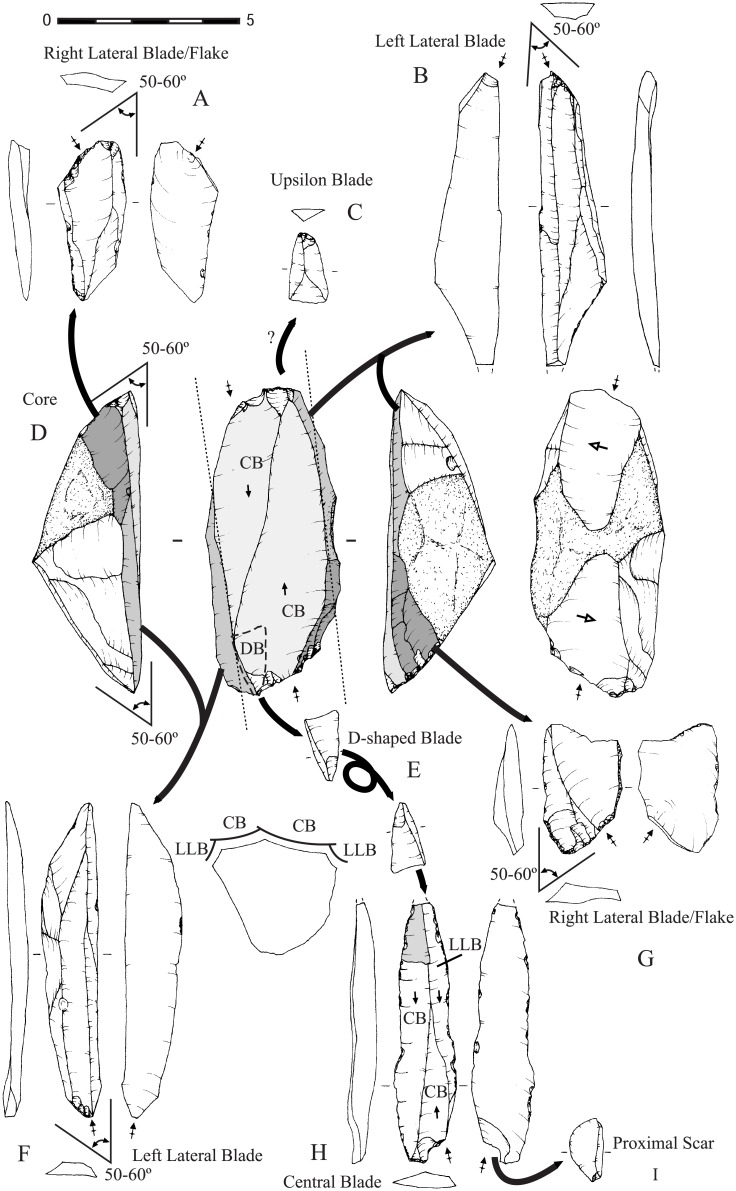
Diagram of the off-set bidirectional blade reduction sequence. For details about the most diagnostic products and traits, see Borrell [37].

In the northern Levant, the regional variable of bidirectional blade technology was first identified in the 1980s [38], termed as ‘naviform method of Douara type’ and systematically described in the 1990s [34], and recently re-defined as the ‘off-set bidirectional strategy’ [37]. This variant is characterized by the narrow and ‘twisted’ working surface and the specific way it is managed. Most characteristic is the detachment of targeted central blades without the removal of ‘upsilon’ or right lateral blades. The direction of flaking is slightly off-set in relation to the longitudinal axis of the core, beginning from the right side of the core platform to the opposite side of the distal end of the core, while the arrises of the last central blade are reused as if it was a lateral blade-scar (Fig 7). In this way, management of the volume is designed to obtain a high ratio of productivity of standardized pointed central blades.

**Fig 8:**
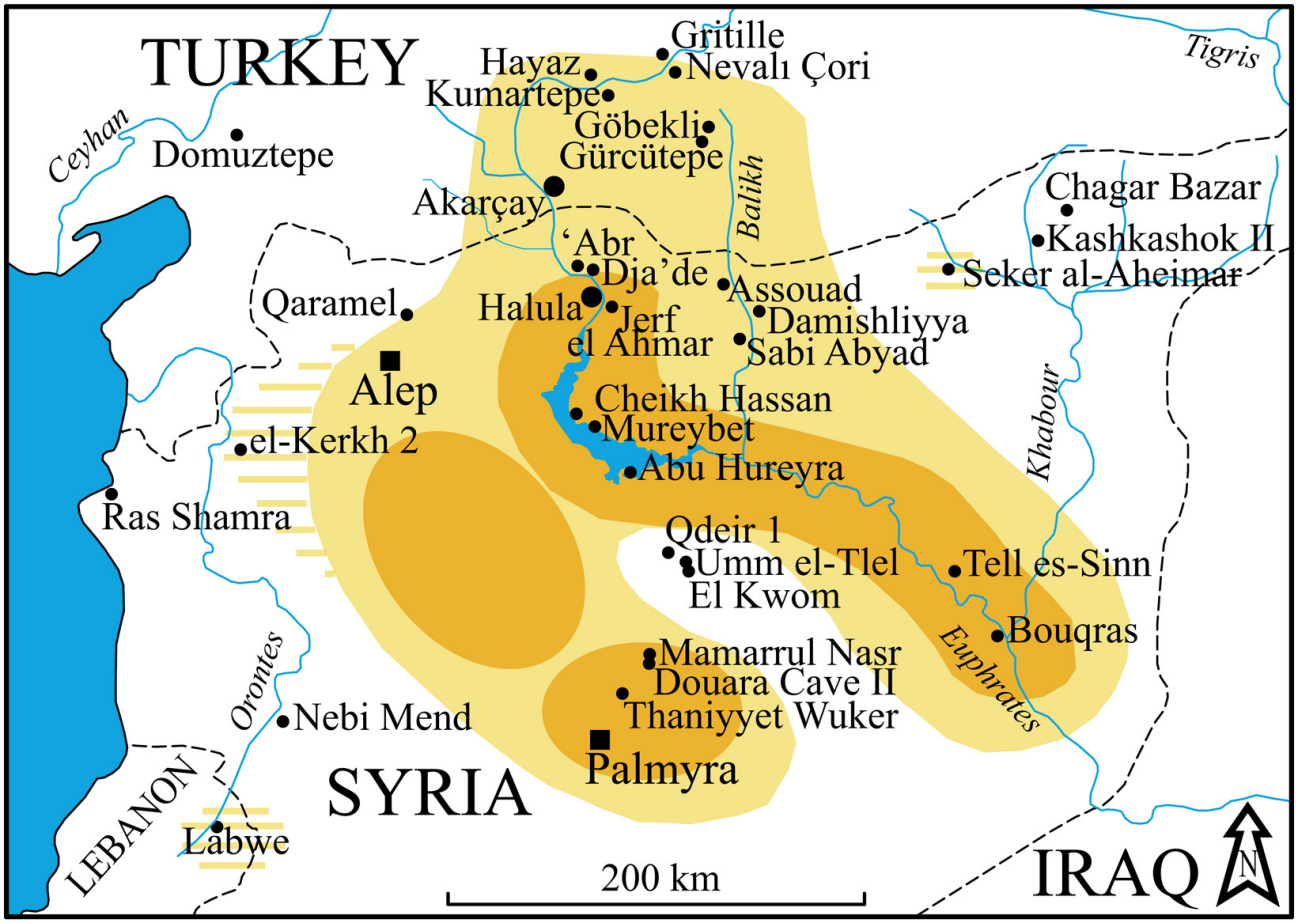
The spatial distribution of the off-set bidirectional strategy in the northern Levant. Core areas and the area of circulation of its products during the Middle/Late PPNB. The earlier settlements are also marked on the map in order to show that they are all located within this same area.

Both the blades and the cores show highly diagnostic traits (e.g. the proximal part of the blades is twisted to the right and the right edge displays a proximal scar on the ventral side that partly removes the butt and the bulb). Thanks to these diagnostic features it has been possible to define the temporal and spatial distribution of this variant in the northern Levant, constituting a specific techno-complex [26]. Two core regions, where massive use of the off-set bidirectional strategy is attested, have been identified: the lower part of the middle Euphrates (at the large sites such as Halula, Abu Hureyra and Bouqras) and in the central Syrian Desert (workshops and campsites such as Douara Cave 2 or Mamarrul Nasr 2) [26,37] (Fig 8). Artefacts produced in the core regions, mostly transformed into projectile points, were widely distributed in other regions such as the upper part of the middle Euphrates valley (Akarçay), Balikh valley (Sabi Abyad and Gürçütepe), the Khabur valley (Seker al-Aheimar) and the Orontes valley (Ain el-Kerkh 2). The exchange of products did not lead to the diffusion of the off-set bidirectional strategy to these regions.

The off-set bidirectional strategy flourished in the Euphrates valley between *ca*. 9,800 until 9,000 cal BP, while in the central Syrian Desert its dating is still debated (either contemporary with the Euphrates valley or slightly later, *ca*. 9,000–8,500 cal BP) [37,39]. The circulation of products from the Euphrates valley to the neighbouring regions is also dated to the same time span. Comparison with the bidirectional blade technology at earlier sites in the middle Euphrates valley like Dja’de, Mureybet, Jerf el Ahmar, Cheikh Hassan or other nearby sites like ‘Abr 3 and Qaramel, shows no connection with the later appearance of the off-set bidirectional strategy. Likewise, no similarity can be seen with the lithics of other earlier sites located further to the north such as Göbekli or Nevalı Çori, nor to the west in the Early PPNB layers at Ain el-Kerkh.

To conclude, the off-set bidirectional strategy was practiced in the middle Euphrates valley no earlier than ~9,800 cal BP, strongly associated with the newly founded settlements and without any apparent predecessors or parallels in the northern Levant. Its sudden appearance accompanies the break in settlement patterns in the middle Euphrates valley, of *ca*. 10,200–9,800 cal BP, indicating a shift in bidirectional blade production strategies from the PPNA and Early PPNB to the Middle and Late PPNB [26].

### Origins and evolution of agricultural practices in the Levant

Near Eastern agriculture is thought to have started with a founder-crop group of seven cereal and pulse grain plant species consisting of diploid einkorn wheat (*Triticum monococcum* L.), tetraploid emmer wheat (*T*. *turgidum* L.), barley (*Hordeum vulgare* L.), lentil (*Lens culinaris* Medikus), pea (*Pisum sativum* L.), chickpea (*Cicer arietinum* L.), and bitter vetch (*Vicia ervilia* L.).

The current debate about exactly how, when and where agriculture first began could be summarized as two alternatives. The ‘core area one event’ model suggests a geographically focused (southeastern Turkey and northern Syria) short-episode domestication for each plant species and for the founder crops package as a whole, from where the domesticated plants diffused throughout the entire Levant. This model stresses the role of highly conscious human action and knowledge-based choice of species and selection of specific genotypes [3,22,40]. The alternative model, the ‘geographically diffused protracted’ domestication model, suggests a long process of domestication of single plant species, the unconscious nature of plant domestication and that plants were domesticated in different places (centres or sub-centres) in the Levant [7,9,25,41]. In terms of chronology and the nature of the process, in the middle Euphrates valley and neighbouring regions, authors proposing the alternative model recognize indications in favour of pre-domestic cultivation beginning at ~11,500 cal BP in sites such as Jerf el Ahmar [8] and later at Dja’de, Ain el-Kerkh and Nevalı Çori. In this interpretation, what defines pre-domestic cultivation is the appearance of large-size seeds and/or presence of a minority of domesticated-type non-shattering rachises, reflecting the process referred to as the ‘domestication syndrome’. Within this approach the earliest morphologically domestic cereals found in this area date to about 10,200–10,000 cal BP [9,16], considered the starting point for a fully domesticated production economy [24].

The opposing view argues that the differences between domesticated plants and their wild progenitors defined as the ‘domestication syndrome’ indicates a pristine conscious domestication within a short time, while pre-domestic cultivation should be considered as intensification of harvesting by foragers. Accordingly, traits used to define morphologically domestic seeds in the geographically diffused protracted model *ca*. 10,200–10,000 cal BP, showing a phenotypic continuum between wild and domesticated gene pools, mostly reflect post-domestication diversification or crop evolution [42]. In sum, what is considered pre-domestic cultivation in one model corresponds to pristine domestication in the other, and this obviously translates to a remarkable chronological discrepancy in ascertaining the timing of the origins of agricultural systems in southwestern Asia.

**Fig 9:**
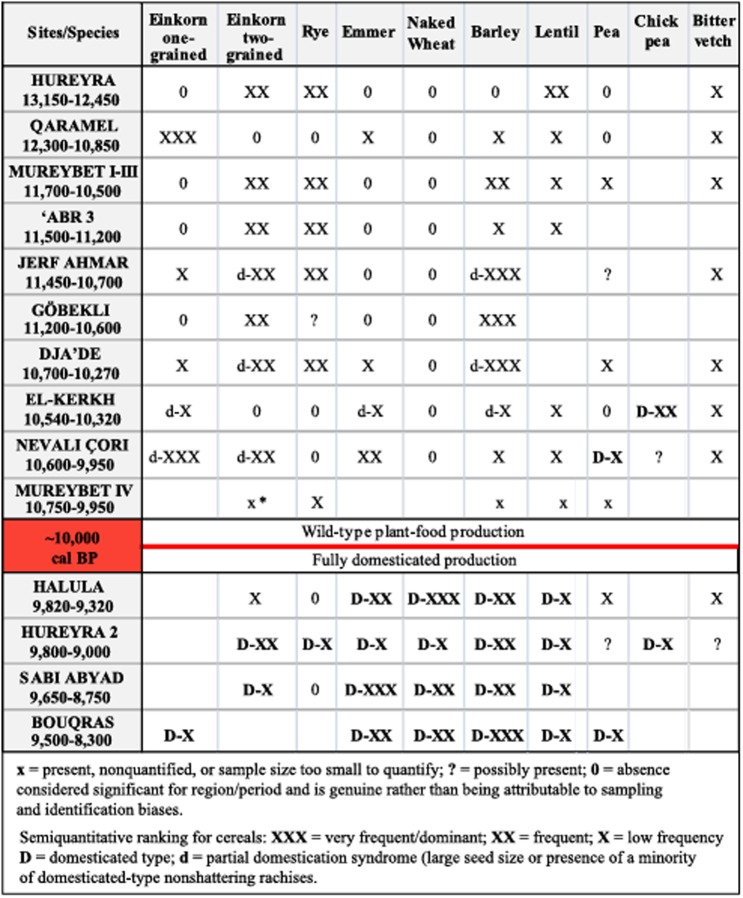
Archaeobotanical record. Evidence for crops and wild progenitors in the Euphrates valley and neighbouring regions/study area (modified from ref. [24]). The chronological time span of each site has not been altered from that on the original chart but adapted to cal BP. *Note that, in this summary authors did not consider data on morphologically domestic two-grained einkorn at Mureybet IV conclusive enough to be included, while it has been in other publications [9].

This debate is far from being resolved and it is beyond the aims of this paper to conclude which approach is more valid. Our multi-proxy approach integrating a large set of radiocarbon dates, settlement history and other aspects of the archaeological record may throw some light on a few aspects of the above-mentioned discussion. The critical examination of the chronology of the sites where an abundance of seeds of domesticated cereals have been recovered—in the protracted approach—shows that there is no solid evidence for the contention that the earliest morphologically domestic cereals are dated to the time prior to *ca*. 10,200–10,000 cal BP. Morphologically domestic cereals recovered in Halula, Abu Hureyra, Bouqras and Sabi Abyad are attributed to the middle PPNB period (10,200–9,600 cal BP) but, as mentioned above, the ^14^C dates demonstrate that none of these was occupied during the 10,200–10,000 cal BP time slot as they were founded around ~9,800 cal BP or later. This means that, according to the available data, in this model the transition from reliance on wild-type cereals to a dominance of the domesticated-type occurred some time between 10,000 and 9,800 cal BP (Fig 9) a couple or more centuries after a marked decline of the archaeological signal associated with a major cultural transformation in the region. These results do not seem to affect the basic aspects of the core area model, which is that pristine domestication occurred slightly after the mid 11^th^ millennium cal BP in the region. However our multi-proxy approach has identified a chronological gap in the settlement history of the region, between the sites where authors consider that pristine domestication occurred and the later large sites, whose economy is considered the results of post-domestication crop evolution. This observation suggests a major shift in agricultural practices between the two groups of settlements. The later settlements represent a new stage in the development of agricultural systems. These communities were fully immersed in an agricultural way of life where domesticated plants had been widely and successfully integrated into their economic systems with a far more complex agro-pastoral economy than was the case with the older settlements. In this sense, it is also significant that discussion about the proportions of wild versus domesticated remains in the archaeobotanical assemblages from the selected Neolithic sites [43] seems to be restricted mainly to the earlier sites, while both models agree that in the later large settlements domestication is not in doubt, morphologically domestic seeds are predominant and naked wheats are present.

In conclusion, a major shift in early agricultural practices, whether the earliest stages can be considered pre-domestic cultivation or pristine domestication, is reported between the old settlements and the later settlements such as Halula, Bouqras and Abu Hureyra. This poorly understood transition in agricultural practices fully coincides with a break in the settlement patterns in the middle Euphrates valley and the drastic drop of the archaeological signal in the region dated to *ca*. 10,200–9,800 cal BP.

### Palaeoclimate record of the early Holocene and cultural change

Following the Younger Dryas event ~11,600 cal BP, the onset of the Holocene in southwest Asia was marked by a rapid climatic shift towards warmer, generally wetter but seasonally dry conditions [10]. Grasslands reached their greatest extent during the early Holocene, and the fire regimes changed markedly due to an increase in biomass [44]. In this sense, the Levant and Eastern Mediterranean regions were not an exception when, during the first half of the Holocene, they experienced a climate regime significantly wetter than today, indicated by the coherent marine and terrestrial isotope and other records in this region [45–49]. The geochemical, isotopic and biological indicators in cores from Eski Acıgöl and Van lakes in Turkey, show higher water levels and lower salinity during the early Holocene when compared to the late Holocene [50,51]. Elevated rainfall is also evidenced on land by increases in Pistacia and oak in the Ghab and Hula pollen records [52]. The southward migration of the Negev Desert boundary [53] and meandering streams in southern Jordan [54] reflect similar conditions. Oxygen-isotope records from a Soreq Cave speleothem demonstrate a shift towards warmer and wetter conditions [55]. The Eastern Mediterranean marine records (cores 9501 and 9509), indicate enhanced rainfall [49], and regional proxies support the interpretation that the early Holocene sapropel S1 period (~9,500–7,000 cal BP) was characterized by enhanced rainfall in the Eastern Mediterranean basin.

The climatic change towards a warmer early Holocene involved a substantial, often abrupt, rearrangement of terrestrial ecosystems, an increase in rainfall and probably affected the availability of food plants. This transformation provided new challenges and resource opportunities for human groups, and has been considered a contributing factor to the emergence of agriculture in the Near East [11,12]. After 7,000 cal BP, the trend changed and climatic conditions in the Eastern Mediterranean region came to resemble those at the present time [56], although some records in the northern Levant show notable discrepancies [57].

Despite the general long-term trends (warmer and wetter during the early Holocene and more arid from 7,000 onwards), climate in the Eastern Mediterranean and the North Atlantic was disrupted by quasi-periodical global cold-dry RCC (Rapid Climate Change) during the Holocene, as observed in a range of palaeoclimate proxies [58–63]. These relatively short isotopic events have also been recorded in regional proxies [49,56], although not all the events and not in all the regional proxies [47,56], indicating that Eastern Mediterranean climate was unstable during the Holocene [56], as in the case of the North Atlantic [58]. It is difficult though to precise the real impact of such RCCs over terrestrial ecosystems during the early Holocene in the different subregions of the Levant as its highly variable climate is affected by a wide range of influences, often involving complex interaction between the circulation induced by ‘remotely driven’ processes (e.g. the North Atlantic storm track or tropical convection) and ‘local’ features (e.g. orography, land—sea contrast) [56]. Furthermore, the Levant is under the influence of two different systems; a southern one strongly influenced by the River Nile input and the northern mostly under the influence of the North Atlantic/Mediterranean climate system [49]. Palaeoclimatic reconstruction for the period is especially problematic in the northern Levant and the Euphrates valley due to the lack of information for the region, making it difficult to determine the exact timing and duration of each RCC and the severity of the effects (changes in temperatures, intensity of seasonality and/or the amount of rainfall). However it is reasonable to assume that global RCCs had a profound impact on the climate, and precipitation in particular, in the northern part of the Levant, as most of the storm tracks that reach the region have a North Atlantic origin [46,64].

Despite this remarkable shortage of local data, the 8.2, 5.2 and 4.2 cal ky BP climate events have raised much attention in Near Eastern archaeology [65–71]. Much attention has thus been dedicated to inferring potential social adaptations and fluctuating population dynamics in the archaeological record, which ultimately led to the diffusion of the Neolithic to southern Europe, as related to the widespread droughts affecting late prehistoric (8.2 cal ky BP) and early historic cultures (5.2 and 4.2 cal ky BP) during each RCC in the Levant.

**Fig 10:**
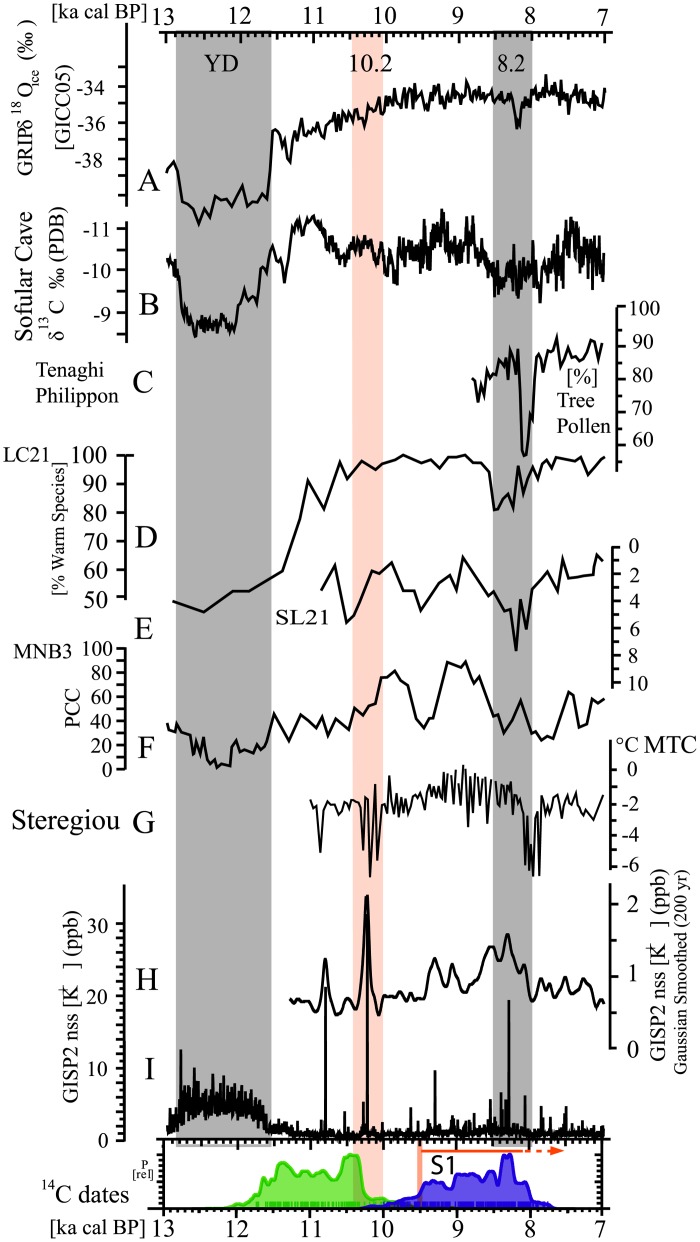
Palaeoclimate proxies from both the Eastern Mediterranean and the North Atlantic. Note the synchrony between 1) the 10.2 cal ky BP RCC and the marked decrease in the archaeological signal in the northern Levant, and 2) the boost observed in the archaeological signal and the beginning of the early Holocene sapropel S1 period (modified from ref. [70]).

The potential impact of the 10.2 and 9.1 cal ky BP abrupt climatic events on human populations at the dawn of agricultural systems has rarely been explored. Multiple climate proxies confirm the existence of a RCC *ca*. 10.2 cal ky BP in the Northern Hemisphere and its cooling effect on the global annual mean temperature [58,72–75]. This climate anomaly has been interpreted as of comparable magnitude to the following events at 9.1 and 8.2 cal ky BP [63]. Interestingly, the 10.2 cal ky BP event is in synchrony with the marked decrease in the archaeological signal observed in the study region, interpreted here as a clear chronological break in the cultural sequence (Fig 10). It is also remarkable that the cultural and socioeconomic transformation that started shortly after the break *ca*. 9,800–9,700 cal BP and, particularly, the subsequent boost in the archaeological signal observed *ca*. 9,500 cal BP corresponds both chronologically and geographically with the beginning of the early Holocene warmer and more humid sapropel S1 period. In conclusion, the temporal correspondence between global and regional climatic events and the archaeological signal indicates that environmental factors influenced cultural changes and had a regional impact resulting in the near abandonment of the region during the 11^th^ to 10^th^ millennium transition.

## Conclusions

This study has identified a cultural break in the apparent continuity of Neolithic communities in most of the northern Levant *ca*. 10,200–9,800 cal BP. The rich archaeological record of architectural, material culture and symbolic features of the Neolithic societies during the initial stages of food production (PPNA-Early PPNB), recently referred to as Early Pre-Pottery Neolithic [24], does not reflect a direct cultural continuity with the later large permanent settlements that emerged around 9,800–9,700 cal BP in the same region (e.g. Halula, Bouqras). These mega-villages, larger than 5 hectares in size, relied on fully domesticated plant production, representing a substantial economic change when compared with earlier sites. They mark a profound socio-economic and cultural transformation and a real ‘point-of-no-return’ for the Neolithic Revolution in the Levant.

The occupational hiatus and cultural break coincide with the rapid cooling of the Northern Hemisphere generally attributed to the 10.2 cal ky BP event. The rapid subsequent socio-economic changes associated with a significant boost in the archaeological signal in the region is in synchrony with the beginning of the warmer and more humid sapropel S1 period (~9,500 cal BP). Such synchrony indicates a correlation between human population dynamics and climate-driven changes in terrestrial ecosystem variability in the region, in the same line as concluded by other researchers [68,76]. It seems that the 10.2 cal ky BP event influenced the drastic weakening of the archaeological signal by disturbing the delicate balance of human-environment interaction among the incipient agrarian populations that had survived for over a thousand years. The abrupt global cold event might have affected the growth of wild plants and predictability of food resources, demanding a better understanding of the process of plant domestication and the conditioning of the terrain. The changing and challenging climate made it difficult for the incipient agrarian populations, which according to some authors might already had been suffering intra societal conflicts [77], to maintain the crop yields needed to sustain a growing and sizable population, precipitating a collapse of widespread occupation from ~10,300 cal BP.

Local communities might have migrated to regions with more favourable conditions or switched to alternative subsistence strategies and possibly mobile lifestyles which emit a weaker archaeological signal. With the eventual amelioration of climate conditions after the 10.2 cal ky BP event, anthropogenic activity reacted positively indicating a potential change in the demographic profile and population growth in the northern Levant, interestingly associated with an agricultural way of life where domesticated plants were widely integrated into their economic systems with a far more complex agro-pastoral economy. This trend started at *ca*. 9,800 cal BP and was consolidated from 9,500 cal BP onwards, during the sapropel S1 period, when climate in the Eastern Mediterranean was characterized by warmer temperatures and enhanced rainfall.

Finally, the identification of a break in the Neolithization process and the positive correlation between human population dynamics and early Holocene climate variability represents a new scenario that is rarely considered in the current hypotheses concerning the origins and consolidation of agriculture and herding in the Levant. Further detailed research concerning regional environmental conditions and population dynamics before and after the hiatus will be crucial to reconstruct the sequence of events that led to the origins and consolidation of agriculture in the Levant. It is also critical for understanding human responses to environmental changes in environments where social vulnerability to climate change is most pronounced, generating new insights into the environmental factors that influenced cultural changes in Levantine prehistory.

## Supporting Information

S1 Table List of radiocarbon dates covering a time span between 11,700–8,700 cal BP. (XLSX)
